# Unnoticed in the tropics: phylogenomic resolution of the poorly known arachnid order Ricinulei (Arachnida)

**DOI:** 10.1098/rsos.150065

**Published:** 2015-06-24

**Authors:** Rosa Fernández, Gonzalo Giribet

**Affiliations:** Museum of Comparative Zoology, Department of Organismic and Evolutionary Biology, Harvard University, 26 Oxford Street, Cambridge, MA 02138, USA

**Keywords:** *Ricinoides*, *Pseudocellus*, *Cryptocellus*, transcriptomics, vicariance, Gondwana

## Abstract

Ricinulei are among the most obscure and cryptic arachnid orders, constituting a micro-diverse group with extreme endemism. The 76 extant species described to date are grouped in three genera: *Ricinoides*, from tropical Western and Central Africa, and the two Neotropical genera *Cryptocellus* and *Pseudocellus*. Until now, a single molecular phylogeny of Ricinulei has been published, recovering the African *Ricinoides* as the sister group of the American *Pseudocellus* and providing evidence for the diversification of the order pre-dating the fragmentation of Gondwana. Here, we present, to our knowledge, the first phylogenomic study of this neglected arachnid order based on data from five transcriptomes obtained from the five major mitochondrial lineages of Ricinulei. Our results, based on up to more than 2000 genes, strongly support a clade containing *Pseudocellus* and *Cryptocellus*, constituting the American group of Ricinulei, with the African *Ricinoides* nesting outside. Our dating of the diversification of the African and American clades using a 76 gene data matrix with 90% gene occupancy indicates that this arachnid lineage was distributed in the South American, North American and African plates of Gondwana and that its diversification is concordant with a biogeographic scenario (both for pattern and tempo) of Gondwanan vicariance.

## 1. Introduction

**Figure 1.**
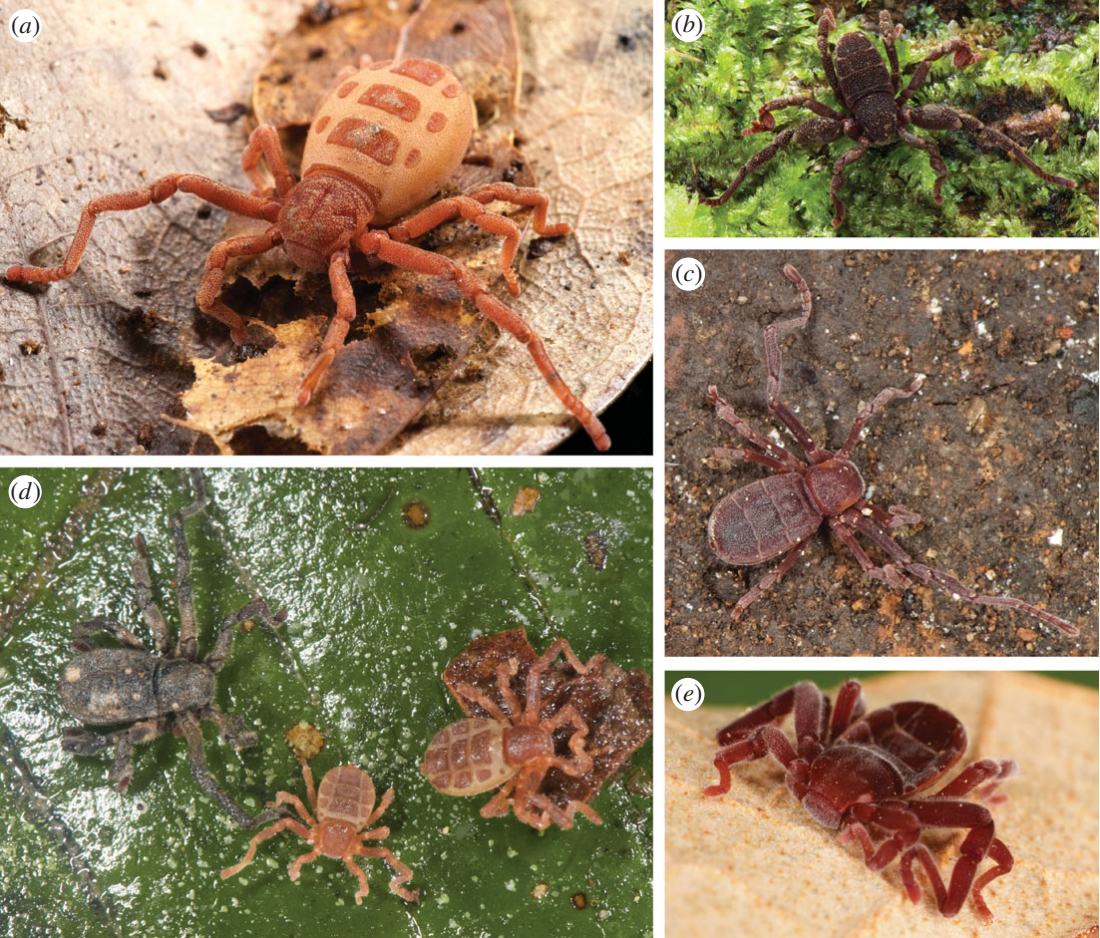
(*a*) Nymph of *Ricinoides atewa* from Asiakwa, Ghana (specimen courtesy of P. Naskrecki) (MCZ IZ-130074). (*b*) Male *Ricinoides karschii* from Campo Reserve, Cameroon, 18.vi.2009 (MCZ IZ-130083). (*c*) Male *Pseudocellus pearsei* from Grutas Tzabnah, Yucatán, Mexico, 23.ix.2011 (MCZ IZ-16426). (*d*) *Cryptocellus becki*, female and two nymphs, from Reserva Ducke, Amazonia, Brazil, 18.v.2012 (MCZ IZ-130034). (*e*) Female of an undescribed *Cryptocellus* sp. from Isla Colón, Bocas del Toro, Panama, 18.iii.2014 (MCZ IZ-30914).

Ricinulei (originally known as Cryptostemmatoidae [1]) are among the most obscure and cryptic of the arachnid orders. They are characterized by having in the anterior region of the prosoma a hinged plate, the cucullus, that acts as a hood covering the mouthparts, by a locking mechanism between the prosoma and the opisthosoma (a trait shared with trigonotarbids, an extinct lineage) that can be uncoupled during mating and egg-laying, and by a modified third leg in males for sperm transfer, among other characters. A total of 76 living Ricinulei species are currently accepted [2,3] in three genera: *Ricinoides* Ewing, 1929 from tropical West Africa (from The Gambia to Gabon), *Cryptocellus* Westwood, 1874 from tropical South America and Central America (Guyana to Peru to Honduras), and *Pseudocellus* Platnick, 1980 from North and Central America (southern USA to Panama) [4] (figure 1).

Despite abundant recent taxonomic work (e.g. [2,3,5–10]), and some phylogenetic and biogeographic studies [11], Ricinulei remains an obscure group, as it was in 1964 when Savory [12] stated that ‘the discovery of each new specimen is still something of a zoological triumph’. Seventy-six years ago, Gertsch *et al.* [13] found the first North American Ricinulei and reported that only *ca* 30 specimens were known for the Americas at the time. Ricinulei have remained a neglected and undersampled group of arthropods until the present, and only a few species are known from more than a handful of specimens. In *Cryptocellus*, three species are still only known by males, six by females only and two only by nymphs [5,6,8,14–21].

With an important fossil record dating back to the Carboniferous [22,23], the phylogenetic position of Ricinulei remains contentious [24]. While virtually all studies have recovered the monophyly of Euchelicerata (=Xiphosura+Arachnida), the monophyly of Arachnida is more controversial and the position of Ricinulei is still unclear, having been recovered as sister group to Acariformes+Parasitiformes [25], Parasitiformes [26], Solifugae [27] or Xiphosura (the later two hypothesis recovered in the same study but with different gene matrices [24]), or recovered as a basal group of Arachnida, excluding Acariformes [28].

As for the phylogenetic relationships within Ricinulei, their internal relationships are virtually restricted to a recent study focusing on the African species belonging to the genus *Ricinoides* [11]. Murienne *et al.* [11] explored the evolutionary relationships between the three currently recognized genera, finding that the African *Ricinoides* was sister group to the American *Pseudocellus*, therefore suggesting that the entire diversification of this arachnid order predated the fragmentation of Gondwana. This biogeographic hypothesis had been previously proposed based on morphological data [29], and may be supported by the presence of fossil Ricinulei from Myanmar [30].

Here, we revisit the internal phylogeny of Ricinulei and present, to our knowledge, the first phylogenomic study of its three extant genera to test the possible paraphyly of the New World clade and to shed further light on the diversification of this cryptic animal group.

## 2. Material and methods

Seventy-nine Ricinulei specimens belonging to the three described genera were collected by sifting leaf litter, or with a Winkler apparatus (table 1). Newly sequenced specimens were collected under permit no. 17 from ARAP (Panama, 27 February 2013), no. 032 from Ministry of Scientific Research and Innovation (Republic of Cameroon, 11 March 2009) and no. 369419 from IBAMA (Brazil, 5 June 2012). We sequenced the mitochondrial marker cytochrome *c* oxidase subunit I (COI) to check the main mitochondrial groups in order to direct transcriptome sequencing efforts, as preliminary results suggested the existence of a high genetic variability within two of the three genera (table 1). Total DNA was extracted from a single leg of each animal using Qiagen's DNEasy^®^ tissue kit. The COI gene was sequenced as described in Murienne *et al.* [11]. The sequence-editing software Geneious v. 6.1.3 [31] was used to read the chromatograms obtained from the automatic sequencer, to assemble both strands for each overlapping fragment and to edit the sequence data. Although alignment was trivial, sequences were aligned in MUSCLE through the online server of EMBL-EBI [32].

**Table 1.** Specimens sequenced for the COI marker. (DNA number, MCZ voucher number, repository, species, country, locality, coordinates and GenBank accession numbers are indicated.)

DNA no.	MCZ voucher	repository	species	country	region	latitude	longitude	accession no. COI
DNA107037	IZ-130034		*Cryptocellus becki*	Brazil	Amazonas, Manaus, Reserva Florestal Adolpho Ducke	−2.93347	−59.97107	KR180414
DNA107038	IZ-130035		*Cryptocellus becki*	Brazil	Amazonas, Manaus, Reserva Florestal Adolpho Ducke	−2.93347	−59.97107	KR180421
		INPA-RI 0093	*Cryptocellus* cf. *becki*	Brazil	Amazonas, BR-319, Taboca, Módulo 3 de Pesquisa do PPBio, Trilha N, Parcela 1500			KR180398
DNA107039	IZ-130037		*Cryptocellus iaci*	Brazil	Roraima, Barreira Branca, Comunidade Caicubí, Rio Jufarí, Municipalidade Caracaraí, Arquipélago de Mariuí e Baixo Rio Branco, Médio Rio Negro	−1.02897	−62.08722	KR180415
DNA107040	IZ-130038		*Cryptocellus iaci*	Brazil	Roraima, Pupunha, Comunidade Caicubí, Rio Jufarí, Municipalidade Caracaraí, Arquipélago de Mariuí e Baixo Rio Branco, Médio Rio Negro	−1.01113	−62.11409	KR180416
DNA105542			*Cryptocellus* cf. *leleupi*	Ecuador	Jatun Sacha Foundation, Upper Napo River, Napo Province			KR180410
DNA102710			*Cryptocellus peckorum*	Colombia	Track to Calderón, Km 22 N of Leticia, Departamento del Amazonas	−4.37888	−69.99027	KR180412
DNA102711	IZ-130028		*Cryptocellus peckorum*	Colombia	comunidad Moniya Amena, Km 9.5 N of Leticia, Departamento del Amazonas	−4.12	−69.92222	KR180411
DNA102698			*Cryptocellus* sp.	Costa Rica	Limon Province, Cahuita Limon, Reserva Biologica Hitoy Cerere	9.67167	−83.025	KR180405
DNA102712			*Cryptocellus peckorum*	Colombia	Track to Calderón, Km 22 N of Leticia, Departamento del Amazonas	−4.04472	−69.98972	JX951321
DNA102713			*Cryptocellus peckorum*	Colombia	Track heading W off Km 13.5 N of Leticia, Departamento del Amazonas	−4.12027	−69.97527	JX951322
DNA102700			*Cryptocellus* sp.	Costa Rica	Limon Province, Cahuita Limon, Reserva Biologica Hitoy Cerere	9.67167	−83.025	KR180406
DNA102701			*Cryptocellus* sp.	Costa Rica	Limon Province, Cahuita Limon, Reserva Biologica Hitoy Cerere	9.67167	−83.025	KR180407
DNA102702			*Cryptocellus* sp.	Costa Rica	Puntarenas province, Cajon, Loc. Curré, Close to River Caño Blancal	8.96278	−83.41583	KR180399
DNA102703			*Cryptocellus* sp.	Costa Rica	Puntarenas Province, Peninsula de Osa, Agua Buena de Rincón. Fundación Neotrópica	8.70056	−83.51306	KR180400
DNA103735	IZ-80067		*Cryptocellus* sp.	Costa Rica	13 km SSW Pto. Jimenez, Puntarenas	8.40667	−83.32833	JX951410
DNA105541			*Cryptocellus* sp.	Costa Rica	La Selva	9.84666	−83.59624	KR180401
		GH1417	*Cryptocellus* sp.	Costa Rica	Cartago, Parque Nacional Tapantí, Macizo de la Muerte, Sendero Natrural Arboles caídos	9.751	−83.77626	KR180419
		GH1418	*Cryptocellus* sp.	Costa Rica	Cartago, Parque Nacional Tapantí, Macizo de la Muerte, Sendero Natrural Arboles caídos	9.751	−83.77626	KR180420
	IZ-127849	TRS05072702LS06	*Cryptocellus* sp.	French Guiana	Nouragues Field Station, XII Trail 1^°^ forest; leaf litter; Winkler sample	4.08875	−52.67617	KR180413
	IZ-127863	JSC06100704LS07	*Cryptocellus* sp.	Guyana	Upper Takutu–Upper Essequibo: Acarai Mts, nr Romeo's Camp; 264 m; 58^°^56.767^′^ W, 1^°^23.334^′^ N; 7 x 2006; J. Sosa-Calvo; 1^°^ forest; leaf litter; Winkler sample	1.3889	−58.94612	KR180417
	IZ-127864	JSC06101001	*Cryptocellus* sp.	Guyana	Upper Takutu–Upper Essequibo: Acarai Mts, nr Romeo's Camp; 294 m; 58^°^56.789^′^ W, 1^°^23.06^′^ N; 10 x 2006; 1^°^ forest; rotten wood; Winkler sample	1.38433	−58.94648	KR180418
	IZ-83251		*Cryptocellus* sp.	Nicaragua	RN El Musún, 3 km NNW Río Blanco	12.95877	−85.22928	KR180404
	IZ-124839		*Cryptocellus* sp.	Nicaragua	RN Cerro Musún	12.95934	−85.22486	KR180403
	IZ-124836		*Cryptocellus* sp.	Nicaragua	PN Cerro Saslaya	13.76867	−84.98446	KR180402
	IZ-124835		*Cryptocellus* sp.	Nicaragua	PN Cerro Saslaya	13.77005	−84.98072	KR180422
	IZ-127866/ IZ-124833		*Cryptocellus* sp.	Nicaragua	RN Kahka Creek	12.67292	−83.71336	KR180423
DNA102709	IZ-130041	GH0756	*Cryptocellus* cf. *chiriqui*	Panama	Prov. Chiriquí: Reserva Forestal Fortuna, Quebrada Honda, hectare PANCODING inventory	8.75008	−82.23908	KR180409
	IZ-127862		*Cryptocellus* cf. *chiriqui*	Panama	Prov. Chiriqui: Reserva Forestal Fortuna, Quebrada Honda	8.75008	−82.23908	KR180424
	IZ-128904.1		*Cryptocellus* sp. nov.	Panama	Smithsonian Research Field Station, Bocas del Toro	9.35215	−82.25699	KR180408
	IZ-128904.2		*Cryptocellus* sp. nov.	Panama	Smithsonian Research Field Station, Bocas del Toro	9.35215	−82.25699	KR180425
	IZ-89406		*Pseudocellus* sp.	Guatemala	5 km SE Antigua	14.53577	−90.69428	KR180445
	IZ-83165	LLAMA RSA 2008-101	*Pseudocellus* sp.	Guatemala	Cerro Carmona, Finca El Pilar	14.53452	−90.69446	KR180444
	IZ-89422		*Pseudocellus* sp.	Guatemala	4 km S Vol. Atitlán	14.54915	−91.19055	KR180441
	IZ-89536		*Pseudocellus* sp.	Guatemala	5 km NW Morales	15.5107	−88.86094	KR180439
	IZ-89548		*Pseudocellus* sp.	Guatemala	5 km NW Morales	15.51405	−88.86524	KR180440
	IZ-99283		*Pseudocellus* sp.	Guatemala	Refugio El Quetzal	14.56339	−91.18554	KR180442
	IZ-98418		*Pseudocellus* sp.	Honduras	P. N. La Muralla	15.09916	−87.74061	KR180438
	IZ-98424		*Pseudocellus* sp.	Honduras	13 km. E Nuevo Ocotepeque	14.45603	−89.06904	KR180437
	IZ-99190		*Pseudocellus* sp.	Honduras	5 km SE Antigua	14.53862	−90.70488	KR180449
	IZ-99193		*Pseudocellus* sp.	Honduras	Parque Nacional La Muralla	15.09387	−86.73934	KR180443
DNA103734	psWard16029		*Pseudocellus* sp.	Honduras		15.58333	−86.66833	JX951409
DNA102697	IZ-130036	AMNH LP5398	*Pseudocellus gertschi*	Mexico	Estación Biológica UNAM, Los Tuxlas, Veracruz	18.57983	−95.08067	KR180436
	IZ-136272		*Pseudocellus monjarazi*	Mexico	Cueva de San Francisco, Municipio La Trinitaria, Chiapas	16.09971	−92.0469	KR180447
	IZ-136270		*Pseudocellus sbordonii*	Mexico	Dentro de la Cueva de las Abejas, Municipio San Fernando, Chiapas	16.8487	−93.24327	KR180448
	IZ-79891		*Pseudocellus* sp.	Mexico	4 km SE Custepec	15.71018	−92.92887	KR180426
	IZ-79891.1							KR180452
	IZ-79891.2							KR180453
	IZ-79891.3							KR180454
	IZ-79891.4							KR180455
	IZ-79891.5							KR180456
	IZ-79966		*Pseudocellus* sp.	Mexico	Mpio. Angel Albino Corzo, Res. Biosfera El Triunfo, Campamento El Quetzal	15.71205	−92.93504	KR180427
	IZ-79966.1							KR180457
	IZ-80001		*Pseudocellus* sp.	Mexico	Mpio. Angel Albino Corzo, Reserva Biosfera El Triunfo, Campamento El Quetzal	15.71032	−92.93218	KR180428
	IZ-80001.1							KR180458
	IZ-80001.2							KR180459
	IZ-80010		*Pseudocellus* sp.	Mexico	Mpio. Angel Albino Corzo, Reserva Biosfera El Triunfo, Campamento El Quetzal	15.72216	−92.94298	KR180429
	IZ-80010.1							KR180460
	IZ-80010.2							KR180461
	IZ-80010.3							KR180462
	IZ-80010.4							KR180463
	IZ-80010.5							KR180464
	IZ-80022		*Pseudocellus* sp.	Mexico	Mpio. Angel Albino Corzo, Reserva Biosfera El Triunfo, Campamento El Quetzal	15.70997	−92.92914	KR180430
	IZ-80025		*Pseudocellus* sp.	Mexico	Mpio. Angel Albino Corzo, Reserva Biosfera El Triunfo, Campamento El Quetzal	15.70775	−92.93121	KR180431
	IZ-80025.1							KR180465
	IZ-80041		*Pseudocellus* sp.	Mexico	Mpio. Angel Albino Corzo, Reserva Biosfera El Triunfo, Campamento El Quetzal	15.72178	−92.94544	KR180432
	IZ-80041.1							KR180466
	IZ-80091		*Pseudocellus* sp.	Mexico	Mpio. Angel Albino Corzo, Reserva Biosfera El Triunfo, Campamento El Quetzal	15.71115	−92.92832	KR180433
	IZ-80112		*Pseudocellus* sp.	Mexico	Mpio. Angel Albino Corzo, Reserva Biosfera El Triunfo, Campamento El Quetzal	15.72122	−92.93913	KR180434
	IZ-80112.1							KR180467
	IZ-80243		*Pseudocellus* sp.	Mexico	Mpio. Angel Albino Corzo, Reserva Biosfera El Triunfo, Campamento El Quetzal	15.70819	−92.9307	KR180435
	IZ-80243.1							KR180468
DNA103736	IZ-79799		*Pseudocellus* sp.	Mexico	3 km SE Custepec	15.71566	−92.93817	JX951411
DNA103736.4	IZ-79799.4		*Pseudocellus* sp.	Mexico	3 km SE Custepec	15.71566	−92.93817	KR180450
DNA105539	IZ-130046		*Pseudocellus boneti*	Mexico	Cueva de Michapa, Town of Michapa, Morelos	18.70278	−99.49417	KR180446
DNA105539.2	IZ130046.2		*Pseudocellus boneti*	Mexico	Cueva de Michapa, Town of Michapa, Morelos	18.70278	−99.49417	KR180451
DNA104741	IZ-130090		*Ricinoides* cf. *olounoua*	Cameroon	Ototomo Forest, near Ngoumou, Central Province	3.64538	11.29033	JX951412
DNA104742	IZ-130091		*Ricinoides* cf. *olounoua*	Cameroon	Ototomo Forest, near Ngoumou, Central Province	3.64447	11.29107	JX951413
DNA104744	IZ-130092		*Ricinoides* cf. *olounoua*	Cameroon	Ototomo Forest, near Ngoumou, Central Province	3.66153	11.30262	JX951415
DNA104745	IZ-130093		*Ricinoides* cf. *olounoua*	Cameroon	Ototomo Forest, near Ngoumou, Central Province	3.66195	11.30025	JX951416
DNA105538	IZ-130094		*Ricinoides* cf. *olounoua*	Cameroon	Ototomo Forest, near Ngoumou, Central Province	3.64513	11.29078	JX951419
DNA104746	IZ-130083		*Ricinoides karschii*	Cameroon	Campo Reserve, *ca* 25 km South of Kribi, Littoral Prov.	2.74108	9.8818	JX951417
DNA102686	IZ-130085	UPV-EHU 2350	*Ricinoides* cf. *karschii*	Equatorial Guinea	South of Ebom, P.N. de los Altos de Nsork, Aconibe District	1.25278	11.05278	JX951397
DNA102687	IZ-130084		*Ricinoides* cf. *karschii*	Equatorial Guinea	South of Ebom, P.N. de los Altos de Nsork, Aconibe District	1.25278	11.05278	JX951398
DNA102682	IZ-130082	UPV-EHU 2348	*Ricinoides gemmifera*	Equatorial Guinea	Región Continental, P.N. de Monte Alén: Itinerario Pedagógico	1.65806	10.31139	JX951396
DNA104743	IZ-130058		*Ricinoides* cf. *karschii*	Gabon	Reserve du Plateau d'Ipassa, Makokou, Ogooué-Ivindo	0.50639	12.79422	JX951414
DNA104747	IZ-130086		*Ricinoides* cf. *karschii*	Gabon	Reserve du Plateau d'Ipassa, Makokou, Ogooué-Ivindo	0.50448	12.79525	JX951418
DNA102691		AMNH LP4658	*Ricinoides feae*	Guinea-Bissau		12.08156	−14.80103	JX951399
DNA102692		AMNH LP4660	*Ricinoides feae*	Guinea-Bissau		12.08156	−14.80103	JX951400
DNA102693		AMNH LP4661	*Ricinoides feae*	Guinea-Bissau		12.08156	−14.80103	JX951401
DNA102693m			*Ricinoides feae*	Guinea-Bissau		12.08156	−14.80103	KR180469
DNA102694		AMNH LP4662	*Ricinoides feae*	Guinea-Bissau		11.88442	−14.83569	JX951402
DNA102695		AMNH LP4664	*Ricinoides feae*	Guinea-Bissau		12.0025	−14.89053	JX951403
DNA102716		AMNH LP4663	*Ricinoides feae*	Guinea-Bissau		11.88442	−14.83569	JX951407
DNA102720		AMNH LP4659	*Ricinoides* aff. *feae*	Senegal	no. km W of Kedougou along road to Salemata	12.55294	−12.22761	JX951408
DNA106890	IZ-130061		*Ricinoides* sp.	Guinea	Mount Nimba	7.54854	−8.52806	KR180470
DNA106891	IZ-130062		*Ricinoides* sp.	Liberia	Mount Yuelliton	7.57814	−8.6111	KR180471
DNA106892	IZ-130063		*Ricinoides* sp.	Liberia	Bassa village, Eastern Nimba Mountains	7.44024	−8.59182	KR180472
DNA106896	IZ-130067		*Ricinoides* sp.	Liberia	Mount Gangra	7.55736	−8.63608	KR180473
DNA106897	IZ-130068		*Ricinoides* sp.	Liberia	Mount Gangra	7.55736	−8.63608	KR180474
DNA106898	IZ-130069		*Ricinoides* sp.	Liberia	Bassa village, Eastern Nimba Mountains	7.44024	−8.59182	KR180475
DNA106899	IZ-130070		*Ricinoides* sp.	Liberia	Bento waterfall			KR180476
DNA102708		AMNH	*Ricinoides atewa*	Ghana	Asiakwa, Eastern Region	6.25039	1.04039	JX951405

Uncorrected *p*-distances between each specimen were calculated and plotted in a heatmap, and maximum-likelihood (ML) and Bayesian inference (BI) phylogenetic hypotheses were generated with RAxML v. 8.0.24 [33] and MrBayes v. 3.2.3 [34] as implemented in the CIPRES Science Gateway [35]. These analyses highlighted five mitochondrial clades: *Pseudocellus* specimens formed a single clade with less genetic variability than *Cryptocellus* or *Ricinoides*, while the other two genera were subdivided into two clades each, exhibiting high genetic variability (see Results and discussion).

Based on these analyses, five Ricinulei specimens representing the three currently recognized genera and the phylogenetic span of the two more diverse genera (*Cryptocellus becki*, *Cryptocellus* sp. nov., *Pseudocellus pearsei*, *Ricinoides atewa* and *Ricinoides karschii*) were selected for transcriptomic analysis. The transcriptomes of *P. pearsei* and *R. atewa* were recently published by our laboratory [24]. Additional arachnid transcriptomes were used as outgroups [24,36] (see Data accessibility; table 2). Note that *Cryptocellus* sp. nov. was collected twice and therefore appears with a different MCZ catalogue numbers in the COI tree (IZ-128904) and the phylogenomic tree (IZ-30913), but they correspond to the same species. Further details can be found in MCZbase, the database of the Museum of Comparative Zoology (http://mcz.harvard.edu/collections/searchcollections.html).

**Table 2.** List of transcriptomes analysed in this study. (Each ricinulei specimen is hyperlinked to its entry in the MCZ database (Harvard University).)

		source	MCZ acc. no.	BioProject (PRJNA)	run (SRR)
outgroups					
*Peripatopsis overbergiensis*	Onychophora	de novo (Illumina HiSeq)	IZ-131372	236 598	1 145 776
*Scutigera coleoptrata*	Myriapoda, Chilopoda	de novo (Illumina HiSeq)	IZ-20415	237 135	1 158 078
*Metasiro americanus*	Chelicerata, Opiliones	GenBank (Illumina GAII)	—	181 108	618 563
*Centruroides vittatus*	Chelicerata, Scorpiones	de novo (Illumina HiSeq)	—	236 506	1 146 578
*Mastigoproctus giganteus*	Chelicerata, Thelyphonida	de novo (Illumina GAII)	IZ-29741	236 514	1 145 698
*Damon variegatus*	Chelicerata, Amplypygi	de novo (Illumina GAI)	IZ-29740	236 494	1 145 694
*Limulus polyphemus*	Chelicerata, Xiphosura	de novo (Illumina HiSeq)	IZ-29738	236 515	1 145 732
*Liphistius malayanus*	Chelicerata, Araneae	de novo (Illumina HiSeq)	IZ-29742	236 495	1 145 736
*Ixodes scapularis*	Chelicerata, Parasitiformes	GenBank (whole genome)	—	—	—
*Tetranychus urticae*	Chelicerata, Acariformes	GenBank (whole genome)	—	—	—
*Synsphyronus apimelus*	Chelicerata, Pseudoscorpiones	de novo (Illumina HiSeq)	—	236 503	1 146 578
*Eremobates* sp.	Chelicerata, Solifugae	de novo (Illumina GAII)	IZ-49755	236 507	1 146 672
Ricinulei					
*Pseudocellus pearsei*		de novo (Illumina HiSeq)	IZ-16426	236 504	1 146 686
*Ricinoides atewa*		de novo (Illumina HiSeq)	IZ-130073 (see also IZ-130074)	236 505	1 145 743
*Ricinoides karschii*		de novo (Illumina HiSeq)	IZ-130083	281 072	1 972 991
*Cryptocellus becki*		de novo (Illumina HiSeq)	IZ-136532 (nymph)	281 078	1 979 416
*Cryptocellus* sp. nov.		de novo (Illumina HiSeq)	IZ-30913 (female)	281 669	1 982 218

Total RNA was extracted with a standard trizol-based method using TRIzol (Life Sciences). After total RNA precipitation, mRNA purification was done with the Dynabeads mRNA Purification Kit (Invitrogen) following manufacturer's instructions. Quality of mRNA was assessed with a pico RNA assay in an Agilent 2100 Bioanalyzer (Agilent Technologies), and quantity was measured with a RNA assay in a Qubit fluorometer (Life Technologies). cDNA libraries were constructed in the Apollo 324 automated system using the PrepX mRNA kit (Wafergen). Concentration of the cDNA libraries was measured through a dsDNA high-sensitivity (HS) assay in a Qubit fluorometer (Invitrogen). Library quality and size selection were checked in an Agilent 2100 Bioanalyzer (Agilent Technologies) with the HS DNA assay. All samples were sequenced in an Illumina HiSeq 2500 platform with paired-end reads of 150 bp at the FAS Center for Systems Biology, Harvard University.

Demultiplexed Illumina HiSeq 2500 sequencing results, in FASTQ format, were retrieved, each sample being quality-filtered according to a threshold average quality score of 30 based on a Phred scale and adaptor trimmed using Trimgalore! 0.3.3 [37]. Ribosomal RNA and mitochondrial DNA were filtered out via Bowtie v. 1.0.0 [38]. Strand specific de novo assemblies were done individually in Trinity [39] using paired read files, a strand specificity flag and path reinforcement distance enforced to 75. Raw reads have been deposited in the National Center for Biotechnology Information Sequence Read Archive database (table 2). Redundancy reduction was done with CD-HIT-EST [40] in the raw assemblies (95% global similarity). Resulting assemblies were processed in TransDecoder [39] to identify candidate open-reading frames (ORFs) within the transcripts. In order to remove the variation in the coding regions of our assemblies due to alternative splicing, closely related paralogs and allelic diversity, predicted peptides were then processed with a further filter to select only one peptide per putative unigene, by choosing the longest ORF per Trinity subcomponent with a Python script. Peptide sequences with all final candidate ORFs were retained as multifasta files. We assigned predicted ORFs into orthologous groups across all samples using OMA stand-alone v0.99y (orthologous matrix [41]). All-by-all local alignments were parallelized across 100 cores of a single compute node, implementing a custom Bash script allowing for execution of independent threads with at least 3 s between each instance of OMA to minimize risk of collisions. Further details and protocols are described elsewhere [36].

Three different amino acid supermatrices were constructed. First, a large matrix was obtained by concatenating the set of orthogroups containing eight or more taxa, yielding a supermatrix with 2177 genes (supermatrix 1: 50% gene occupancy; 568 293 amino acids). To increase gene occupancy and to reduce the percentage of missing data, a second matrix was created by selecting the orthologues contained in 13 or more taxa (supermatrix 2: 476 genes; 75% gene occupancy, 98 933 amino acids), and a third matrix was built choosing the orthologues present in 16 or more taxa (supermatrix 3: 76 genes; 90% gene occupancy; 12 919 amino acids). ML inference was conducted with PhyML-PCMA (supermatrices 2 and 3) [42] and PhyML implementing the integrated branch length option (supermatrix 3) [43]. Bootstrap support values were based on 100 replicates. We selected 20 PCs in the PhyML-PCMA analyses and empirical amino acid frequencies. Bayesian analysis was conducted with ExaBayes [44] (two runs, three independent Markov chain Monte Carlo, MCMC chains per run) in the three supermatrices. A 50% majority-rule consensus tree was computed from the combined remaining trees from the independent runs. For practical reasons and due to the similar results obtained for the different phylogenetic analysis (see the Results and discussion), in the big supermatrix only a ML analysis was explored (PhyML-PCMA).

To discern whether compositional heterogeneity among taxa and/or within each individual orthologue alignment was affecting phylogenetic results, we further analysed supermatrices 2 and 3 (76 and 476 genes) in BaCoCa v. 1.1 [45]. The relative composition frequency variability (RCFV) values (that measures the absolute deviation from the mean for each amino acid for each taxon) was plotted in a heatmap using the R package gplots with an R script modified from [45].

To investigate potential incongruence between individual gene trees, best-scoring ML trees were inferred for each gene included in each supermatrix under the Protgammalg4 with RAxML v. 8.0.1 [33]. Gene trees were decomposed into quartettes with SuperQ v. 1.1 [46] and a supernetwork assigning edge lengths based on quartette frequencies was inferred selecting the ‘balanced’ edge-weight optimization function, applying no filter; the supernetworks were visualized in SplitsTree v. 4.13.1 [47].

A key aspect of ricinuleid systematics is their tempo of evolution and whether it is consistent with a biogeographic scenario of Gondwanan vicariance, so we used the 76 gene dataset for dating. The fossil record of Ricinulei is impressive considering the current low diversity and restricted distribution, confined to the tropical regions of both sides of the Atlantic. Selden [23] revised the fossil ricinuleids and erected the clade Palaeoricinulei for the extinct species, limiting Neoricinulei to the extant ones. At the time, Palaeoricinulei included several Carboniferous species, the oldest being *Curculioides adompha*, from rocks of the upper Namurian B stage of the Ruhr area, Germany, while the remaining species were Westphalian in age, from the USA and the UK [23]. Subsequently, a species from fossiliferous Cretaceous amber of Myanmar was described [30], which has been recently constrained to the earliest Cenomanian age [48]. The age of 98.79±0.62 Ma can be used as a maximum limit for the burmite (either at or after). Although described as a Palaeoricinulei, we consider that the Myanmar fossil belongs to crown-group Neoricinulei, and we use this age as a constraint for the extant taxa.

As for the outgroups, the split between Onychophora and Arthropoda was dated between 528 Ma (the minimum age for Arthropoda used by Lee *et al.* [49] on the basis of the earliest *Rusophycus* traces) and 558 Ma, used as the root of Panarthropoda [49]. The Siluro-Devonian scutigeromorph centipede *Crussolum* [50,51] constitutes the oldest centipede fossil. We thus apply 418 Ma to the split between *Scutigera* and Chelicerata. We used *Lunataspis aurora*, considered as the oldest xiphosuran (*ca* 445 Ma), to date the split between Xiphosura and Arachnida [52]. The split between Scorpiones and Tetrapulmonata was dated to 418 Ma based on *Proscorpius osborni* [53]; *Proscorpius* is neither the oldest geologically nor the most basal scorpion, but it is one of the best known thanks to numerous well-preserved specimens. The split between Araneae, Thelyphonida and Amblypygi and their respective sister groups was dated at 312 Ma, 411 Ma for Opiliones, 308 Ma for Solifugae and 411 Ma for Acari (see a review in [22]).

Divergence dates were estimated using the Bayesian relaxed molecular clock approach as implemented in PhyloBayes v. 3.3f [54] under the autocorrelated lognormal and uncorrelated gamma models and two independent MCMC chains (10 000–12 000 cycles). For dating, we followed a recent review of the oldest occurrences of each arachnid taxon by Dunlop [22] and employed the conservative approach of using the oldest occurrence of a crown-group to constrain the split from its sister group. The calibration constraints were used with soft bounds [55] under a birth–death prior.

## 3. Results and discussion

**Figure 2.**
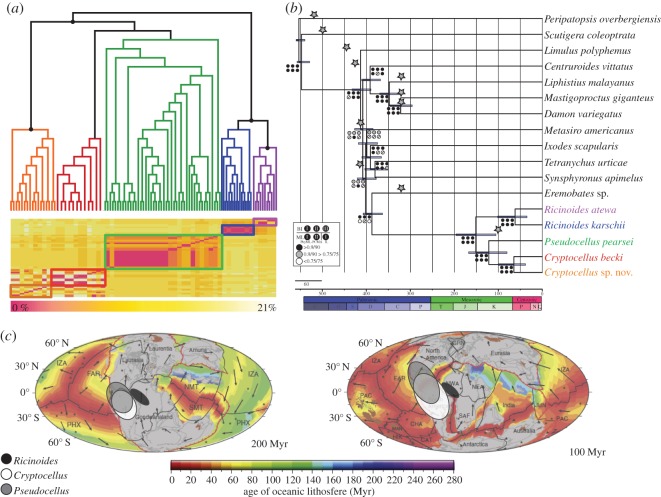
(*a*) Top, maximum-likelihood tree of the COI sequence data (supermatrix 1). Black dots indicate a bootstrap support value higher than 90% (see the electronic supplementary material, figure S1, for further details). Bottom, heatmap of genetic distances between the main lineages of Ricinulei. Ricinulei taxa colour-coded as in (*b*). (*b*) Phylogenomic hypothesis of the evolutionary relationships of Ricinulei. Node support for the different analyses is indicated in each case, as described in the legend. Grey stars indicate fossil calibration points. (*c*) Palaeogeographical reconstruction according to Seton *et al.* [56] at the maximum and minimum ages of the split of the Ricinulei main lineages, as recovered by the molecular dating analysis. Colour bar indicates the age of oceanic lithosphere. The distribution area of the three genera described to date is shown.

Analysis of the COI dataset including 103 specimens clearly identifies the presence of five major Ricinulei lineages, although the COI data fail to find monophyly of *Cryptocellus* (figure 2*a*). These results, even with a much larger sampling of Neotropical species, are not too different from those presented by Murienne *et al.* [11]. These five lineages, however, defined the five clades for which species were selected for the subsequent phylogenomic analyses (figure 1), the focus of the remainder of the discussion.

**Figure 3.**
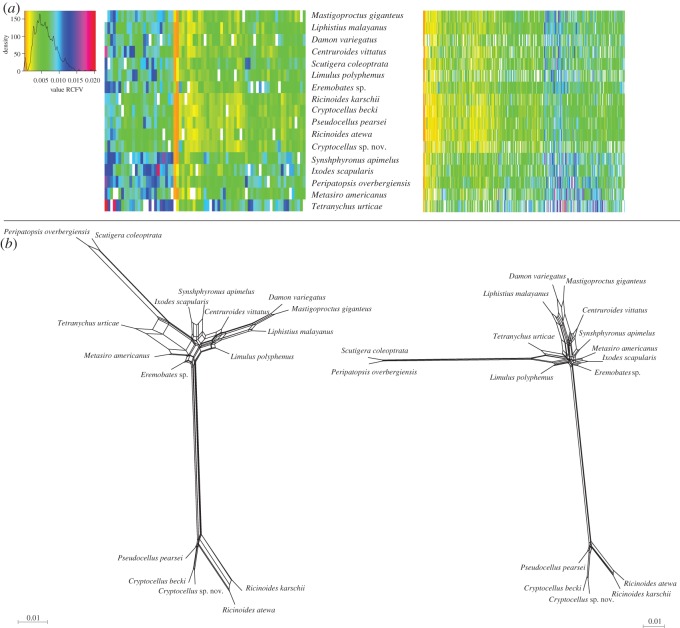
(*a*) Heatmap showing the RCFV values (that measures the absolute deviation from the mean for each amino acid for each taxon) in supermatrices 2 (476 genes, right) and 3 (right, 76 genes). (*b*) Supernetwork visualization of individual gene trees in supermatrices 2 (right) and 3 (left). The lack of reticulation indicates no conflict between individual gene trees.

This is, to our knowledge, the first study addressing the phylogenetic reconstruction of the order Ricinulei beyond the resolution provided by Sanger sequencing. All the recovered phylogenomic trees are concordant and clearly show a split between two major clades: one formed by the African genus *Ricinoides*, and a second one that includes *Pseudocellus* and the two *Cryptocellus* (figure 2*a*), supporting an early split of the Afrotropical and Neotropical species. By contrast, prior work [11] recovered the African *Ricinoides* as sister to the Neotropical *Pseudocellus*. From the three genera, *Pseudocellus* shows more homogeneity than the other two genera in the Sanger-based data analysis, while the African *Ricinoides* and the Neotropical *Cryptocellus* appear to have deep structure with two major clades each (figure 2*b*; [11]). However, the phylogenomic data strongly support monophyly of both *Ricinoides* and *Cryptocellus* (figure 2*a*) and show no conflict at the gene-tree level (figure 3).

Our results are also congruent with early vicariance during the early evolution of extant Ricinulei at the initial breakup of Gondwana. The dating analyses further corroborate the vicariance hypothesis, as we found that the split between *Ricinoides* and the clade formed by *Pseudocellus* and *Cryptocellus* dates back at least to the Early Cretaceous (105–195 Ma), refuting the need of transoceanic dispersal to explain their current distribution (figure 2*b*,*c*), even when considering the Myanmar Cretaceous fossils, as these are probably a sister group to the extant clade and therefore may have diverged much earlier in the Mesozoic. In the South Atlantic, ocean floor extension began within continental South America at 150 Ma, inducing a rift zone between South America and Africa. Spreading extended southward along the South Atlantic ridge with a northward propagation leading to seafloor spreading in the ‘Central’ segment by 120 Ma and in the ‘Equatorial’ segment by 110 Ma. From 100 Ma, the Middle and South Atlantic Ridges were well established and rifting in the interior of Africa ceased at about 85 Ma (figure 2*b*,*c*; [56,57]). These dates are thus concordant with our phylogenomic dating.

Cladogenesis of the Neotropical genera is slightly more recent (from the Late Cretaceous to the Middle Jurassic; 80–167 Ma), but still occurring potentially before the fragmentation of the South American, African and North American plates, reinforcing vicariance as a main force of diversification in Ricinulei (figure 2*c*). The development of the Caribbean is tied to the rifting of the central Atlantic during the break up of Pangea, which extended into the Caribbean during the Triassic to the Early Cretaceous. Spreading along the Central Atlantic Ridge continued into the proto-Caribbean Sea until 100 Ma [56], and the initiation of the Panama–Costa Rica Arc occurred around 80–88 Ma [58]. The reciprocal monophyly of *Cryptocellus* and *Pseudocellus* indicates a possible vicariant model of cladogenesis between these two genera, the former predominantly South American, the latter predominantly Caribbean, Meso-American and North American. Future studies should determine the age of the diversification of *Pseudocellus* and its potential for understanding the palaeogeography of the Caribbean region [59].

Ricinulei constitute a poorly studied arachnid order which once had a broader distribution, including species in southeast Asia [30], but is now restricted to the tropical regions of West Africa and the Americas. Our data however show that this arachnid order has persisted largely unchanged for over 100 Myr, with a conservative phylogenetic pattern able to trace not only old continental movements, but also preserving regional information about the persistence of forests through time [11]. Similar patterns of vicariant diversification are common in other soil-dwelling and saproxylic animal groups originating in Gondwana, including velvet worms [60], centipedes [61] and caecilians [62]. Ricinulei is thus more than just another obscure animal group, and should be studied as a relictual arachnid order with the potential of providing a modern explanation to recalcitrant questions such as ancient Caribbean biogeography.

## Data accessibility

All COI sequences were deposited in GenBank. The accession number for the sequence of each specimen is indicated in table 1. The raw data of the new transcriptomes generated for this study were deposited in the Sequence Read Archive database (SRA) of NCBI. Accession numbers are shown in table 2.
